# Integrin CD11c regulates B cell homeostasis

**DOI:** 10.3389/fimmu.2024.1359608

**Published:** 2024-02-06

**Authors:** Lifei Hou, Yi-Cheng Sin, Yue Chen, Koichi Yuki

**Affiliations:** ^1^Department of Anesthesiology, Critical Care and Pain Medicine, Cardiac Anesthesia Division, Boston Children’s Hospital, Boston, MA, United States; ^2^Department of Anaesthesia and Immunology, Harvard Medical School, Boston, MA, United States; ^3^Department of Biochemistry, Molecular Biology and Biophysics, University of Minnesota, Minneapolis, MN, United States

**Keywords:** CD11c, dendritic cell, B cell, integrin, MIF

## Abstract

CD11c is widely known as a cell surface marker for dendritic cells, but we recently showed that it regulates neutrophil and T cell functions. Because we found that CD11c knockout (KO) mice had lower blood B cell counts, we characterized B cell profile in developmental stages. We found that CD11c KO recirculating and mature B cells was significantly fewer compared with wild type, associated with exaggerated proliferation and apoptosis. Because they did not express CD11c, we sought for the possibility of CD11c-mediated non-intrinsic regulation of B cell proliferation and apoptosis. Here we hypothesized that dendritic cells, major cells expressing CD11c would regulate B cells indirectly. The proteomics of dendritic cells cultured *in vitro* indicated the downregulation of macrophage migration inhibitory factor (MIF). Less MIF was also confirmed by ELISA. Furthermore, plasma MIF level was significantly lower in naïve CD11c KO mice. Because MIF regulates B cell survival, we demonstrated a novel regulatory mechanism of naïve B cells via CD11c.

## Introduction

CD11c/CD18 (CD11c, also called integrin αXβ2) is one of adhesion molecule β2 integrin family members. Although it is reported that it binds to iC3b, intercellular adhesion molecule-1 (ICAM-1) and fibrinogen *in vitro* (1, 2), it is primarily known as a dendritic cell marker *in vivo*. However, we recently reported that CD11c regulates neutrophil maturation and T cell development (3, 4). Thus, it would be of great interest to examine if CD11c also affects other cell types.

B cell development starts in the bone marrow (BM) and matures in the spleen. Heavy (H) and light (L) chain immunoglobulin genes are rearranged at the pro-B and pre-B stages, and complete surface immunoglobulin M (IgM) is expressed on immature B cells (5). Then, they undergo positive and negative selection, mediated by B cell antigen receptor (BCR) to create repertoire of naïve B cells. We previously found that blood B cell counts were significantly lower in CD11c KO mice compared to wild type (WT) mice (6). In this study, we examined how CD11c was involved in regulating naïve B cell number. We found that CD11c KO recirculating and mature B cells was significantly fewer compared with wild type, associated with exaggerated proliferation and apoptosis. Given they did not express CD11c, we sought for the possibility of CD11c-mediated non-intrinsic regulation of B cell proliferation and apoptosis under the hypothesis that dendritic cells, major cells expressing CD11c would regulate B cells indirectly. We demonstrated a novel role of CD11c in our B cell immune system.

## Methods

### Mice

Animal studies were approved by the Institutional Animal Care and Use Committee of Boston Children’s Hospital. Wild type C57BL/6J mice and CD11c KO mice were previously described in our publication (6).

### Flow cytometry analysis

Anticoagulated blood, splenocyte and bone marrow cells were put on ice, blocked with Fc receptor blocker (anti-mouse CD16/32 antibody, clone 93, Biolegend, San Diego, CA), and stained at 4°C with various fluorochrome-conjugated antibodies to surface markers. After washing, cells were fixed for flow cytometry analysis, or were permeabilized and stained intracellularly with fluorochrome-conjugated antibodies using fixation/permeabilization reagents and protocols from BD Bioscience. Fluorochrome-conjugated antibodies were obtained from Biolegend, which include:

APC- or Pacific blue-anti-mB220 (RA3-6B2), FITC- or PE-Cy7- or APC- anti-mCD11C (N418), PE-Cy7-Hamster IgG (HTK888, isotype control for CD11c antibody), PE-anti-mIgM (RMM-1), APC-anti-mIgD (11-26c.2a), FICT-anti-mCD93 (AA4.1), PE-CY7-anti-mCD23 (B3B4), Pacific blue- or APC-anti-mCD45.1 (A20), Pacific blue-anti-mCD45.2 (104). For CD11c intracellular staining, we saturated cell surface CD11c (if any) with unlabeled anti-CD11c monoclonal antibody (N418), and then fixed, permeabilized cells, and stained the intracellular CD11c by fluorescence-labeled anti-CD11c antibody (N418). Cell counting was done by applying Sphero AccuCount beads (ACBP-50; Spherotech Inc, Lake Forest, IL). Data were acquired on a Canto II cytometer (BD Biosciences, Franklin Lakes, NJ) and analyzed using FlowJo software (Tree Star, Ashland, OR). Regarding the anticoagulated blood sample, red blood cells were lyzed after the staining. Regarding splenocyte and bone marrow cell staining, spleen and bone marrow were made into single cell suspension, and then red blood cell were lyzed by red cell lysing buffer (BD Bioscience), followed with staining.

For detection of phospho-Erk1/2, cells were cultured in the complete RPMI-1640 medium containing 10% FCS for 30 min, and then were put in ice, stained with surface markers on ice, washed at 4°C, then fixed at 37°C for 10 minutes by Fix Buffer 1 (cat: 557870), and permeabilized by Perm Buffer III (cat: 558050), and then stained with isotype control (clone: MOPC-21) or anti-phospho-Erk (clone: 20A). Phosphoflow-related reagents and antibodies were purchased from BD Bioscience.

### Proliferation and apoptosis

Proliferation of cells was examined either with Ki67 staining or *in vivo* 5-bromo-2′-deoxyuridine (BrdU) incorporation. Mice were injected intraperitoneally (*i.p.*) with 1 mg of BrdU. To detect BrdU incorporation into bone marrow hematopoietic cells, BD cytofix/cytoper Plus kit (555028; BD Biosciences) and Alexa Fluor 488–conjugated anti-BrdU antibody (3D4; BioLegend) were applied according to the manufacturer’s protocol. Cells were subjected to flow cytometry analysis. For Ki67 staining, cells were intracellular stained with anti-mKi67 antibody (11F6; BioLegend). Apoptosis was detected with intracellular staining of active caspase-3 antibody (559341; BD Biosciences).

### Bone marrow derived dendritic cell culture

Bone marrow (BM) cells were obtained and cultured in 6 well plates in RPMI1640 supplemented with 10% heat inactivated FBS, glutamine, penicillin, streptomycin, GM-CSF (20 ng/mL) and IL-4 (5 ng/mL). At day 3, the culture medium was entirely discarded and replaced with freshly warmed medium containing GM-CSF and IL-4 (3). At day 7, part of cells was subjected to lipopolysaccharide (LPS) 10 µg/mL stimulation for 24 hours.

### BM derived dendritic cell proteomics analysis

#### Sample preparation

Cells were lysed in urea lysis buffer [9M Urea, 50 mM ammonium bicarbonate, and cOmplete™ Protease Inhibitor Cocktail (Roche)], followed by sonication. Proteins were reduced and alkylated with a final concentration of 10 mM tris [2-carboxyethyl) phosphine (TCEP)] (Pierce, Appleton, WI) and 10 mM iodoacetamide (IAA) (VWR, Radnor, PA) in darkness at room temperature for 30 minutes. The reaction was halted by bringing it to a final concentration of 20 mM cysteine and incubating it for 30 minutes at room temperature. After the dilution of urea concentration to 1.5 M, samples were digested with trypsin at an enzyme-to-substrate ratio of 1:50 (w/w) at 37°C overnight, and the digestion was repeated for another 2 hours the next day. Tryptic peptides were desalted with in-house C18 Stage tips packed with Empore C18 membrane (CDS Analytical, Oxford, PA) and dried in SpeedVac (Thermo Scientific, Waltham, MA) before the liquid chromatography-mass spectrometry (LCMS) analysis.

#### LCMS analysis

Peptides were analyzed by nano-flow LCMS analysis using a Dionex Ultimate 3000 RSLCnano coupled to an Orbitrap Fusion mass spectrometry (Thermo Scientific, Waltham, MA) at the Center for Metabolomics and Proteomics at the University of Minnesota. Peptides were dissolved in HPLC buffer A [0.1% formic acid in water (v/v)] and separated on an in-house packed reversed-phase C18 capillary HPLC column (40 cm in length, 100 μm in inner diameter) packed with C18 beads (C18 AQ Dr. Maisch ReproSil-Pur, 120 A pore size, 1.9 um resin size). The peptides were separated with a 90-min gradient from 5% to 90% HPLC buffer B [0.1% formic acid in acetonitrile (v/v)] in HPLC buffer A. Full scan of precursor ions (MS1) was performed in a positive mode with an RF lens at 60% and a resolution of 120K (at 200 m/z) at a mass range of 380 – 1580 m/z. Tandem mass spectra (MS/MS) were acquired using the linear ion trap, employing an isolation window of 1.6 m/z and a high-energy collision dissociation (HCD) energy of 35%. Dynamic exclusion with a mass tolerance of ± 10 ppm and a 30-second exclusion window was set.

#### Data analysis

LCMS raw data were analyzed by MaxQuant searching software (version 1.5.3.12) (7). Peptide and protein identifications underwent filtration at a 1% false discovery rate (FDR) by searching against the UniProt mouse database (downloaded August 18, 2021) in conjunction with the reversed decoy database. Trypsin was used as the digestion enzyme with a maximum of 2 missing cleavages. Cysteine carbamidomethylation was assigned as a fixed modification. Variable modifications include lysine acetylation, methionine oxidation, phosphorylation of serine, threonine, and tyrosine, and protein N terminal acetylation with a maximum number of 3. The diagnostic peaks of lysine acetylation were set as the composition of C(7)H(11)NO and C(7)H(14)N(2)O. Neutral loss of phosphorylation was not assigned in the search. Label-free quantification (LFQ) and matches-between-run functions were enabled for the analysis.

Protein quantification was performed with Perseus software (version 1.6.15.0) (8). Briefly, LFQ values of proteins in each sample were logarithm-transformed, and proteins that were quantified in less than half of the total samples were removed. Imputation of LFQ missing values was performed with normal distribution, and statistical significance of protein abundance changes between groups of samples was achieved with the Student’s t-test (false discovery rate of 0.05).

### Macrophage migration inhibitory factor ELISA

Plasma and cultured supernatants were subjected to measuring macrophage migration inhibitor factor (MIF) levels using MIF ELISA kit (R&D systems, Minneapolis, MN) according to the company’s protocol.

### Reverse transcriptase-quantitative PCR

BM derived dendritic cells (BMDCs) were subjected to RNA isolation using RNeasy Plus kit (74134, Qiagen, Hilden, Germany) according to the manufacturer’s protocol and reserve transcribed using iScript cDNA synthesis kit (Bio-Rad, Hercules, CA). The qPCR assays were performed using SYBR Green. Primers we used were mouse interferon beta: F (5’ -> 3’): CAGCTCCAAGAAAGGACGAA, R (5’ -> 3’): GGCAGTGTAACTCTTCTGCAT; mouse MIF:

F (5’ -> 3’):GCCAGAGGGGTTTCTGTCG; R (5’ -> 3’): GTTCGTGCCGCTAAAAGTCA; mouse GAPDH: F (5’ -> 3’): AGGTCGGTGTGAACGGATTTG, R (5’ -> 3’): TGTAGACCATGTAGTTGAGGTCA.

### Statistical analysis

Data were analyzed as indicated in the corresponding figure legends. Statistical significance was defined as p< 0.05. All the statistical calculations were performed using PRISM5 software (GraphPad Software; La Jolla, CA).

## Results

### Blood, splenic and bone marrow recirculating B cell counts were significantly lower in CD11c KO mice

**Figure 1:**
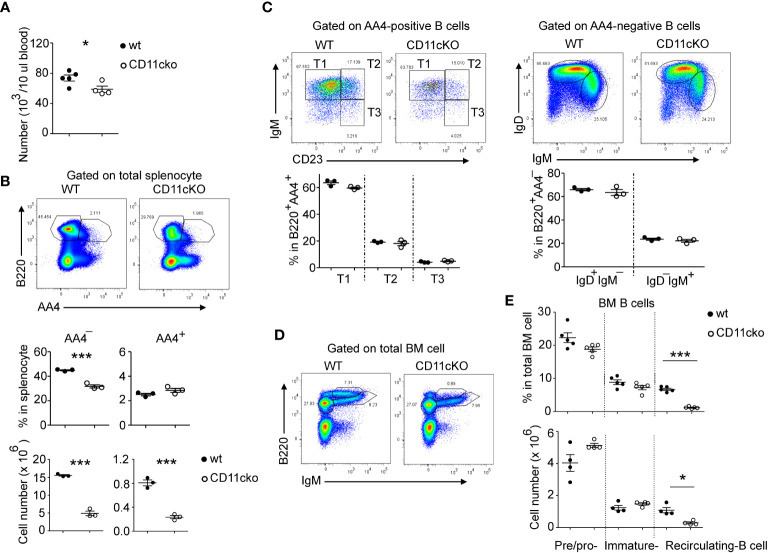
The effect of CD11c on B cell development. **(A)** Comparison of blood B cell counts between WT and CD11c KO mice. **(B)** Comparison of splenic B cell between WT and CD11c KO mice. Up-panel: Representative flow cytometry plots comparing the splenic B cell maturation status between WT and CD11cKO mice. At least five independent experiments were done with similar pattern. Bottom-panel: Statistical analysis. **(C)** Comparison of immature splenic B cells (B220^+^AA4^+^) and mature splenic B cells (B220^+^AA4-) between WT and CD11c KO mice. Up-panel: Representative flow cytometry plots; Bottom-panel: Statistical analysis. **(D)** Representative flow cytometry plots of BM pre/pro-B cells, immature B cells and recirculating B cells. Experiments were repeated at least 3 times with the same pattern. **(E)** Statistical analysis showing both ratio (up-panel) and absolute number (bottom-panel) of BM pre/pro-B cells, immature B cells and recirculating B cells between WT and CD11c KO mice. For **(A)**, bottom-panel in **(B, C, E)**, Data were shown as mean +/- S.E.M. Each symbol represents one mouse. Student t test was used for statistical analysis. * p< 0.05; *** p<0.001. Experiments were repeated at least 3 - 5 times with the same pattern.

We found that CD11c KO mice had significantly lower blood B cell (**Figure 1A**) and splenic B cell counts (**Figure 1B**) compared to WT mice. B cell precursors are produced in the bone marrow (BM), selected by self-tolerance mechanism including clone deletion and receptor editing, and then released into peripheral lymphatic organs, such as spleen, to undergo further maturation and acquire high level of surface IgD and CD23 (9). Since total B cell counts in the blood were significantly less in CD11cKO mice when compared with WT mice, we then studied the B cell maturation status. As shown in **Figure 1B** (middle panel), although the total B cell ratio in spleen was lower in CD11cKO mice, the ratio of immature B cell (B220^+^AA4^+^) was not different between genotypes. In the spleen, those AA4^+^ immature B cell further matures by going through the transitional (T) stage 1 (T1: IgM^+^CD23^-^), T2 (IgM^+^CD23^+^) and T3 (IgM^-^CD23^+^) (10). Further staining of surface IgM and CD23 showed that deficiency of CD11c didn’t affect the transitional stages (**Figure 1C**). Co-staining of IgM and IgD on AA4^-^ matured B cells also confirmed that deficiency of CD11c didn’t affect the maturation status of B cells (**Figure 1C**). Since the B cell counts in the blood were much lower in CD11cKO mice, we then investigated the bone marrow, where B cells were initially generated. BM analysis showed no difference in the number of pre/pro B cells and immature B cells between WT and CD11cKO mice (**Figures 1D, E**). In contrast, the number of recirculating B cells was significantly lower in CD11c KO BM compared to WT BM (**Figures 1D, E**), consistent with the observation of blood and splenic B cell, which can circulate into BM as well (**Figures 1A–C**).

### Recirculating and mature B cells experienced accelerated proliferation and apoptosis

**Figure 2:**
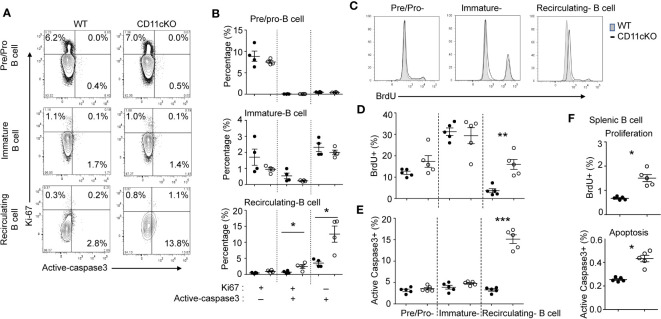
The role of CD11c in B cell proliferation and apoptosis. **(A)** Representative plot of proliferation (Ki-67) and apoptosis (cleaved caspase 3) in BM pre/pro B cells, immature B cells and mature B cells. At least five independent experiments were done with similar pattern. **(B)** Statistical analysis of proliferation and apoptosis of BM pre/pro-B cells, immature B cells and mature B cells probed by Ki67 and cleaved caspase 3 expression, respectively. **(C)** Representative histogram of proliferation of BM pre/pro-B cells, immature B cells and mature B cells probed by BrdU incorporation. At least five independent experiments were done with similar pattern. **(D)** Statistical analysis of proliferation of BM pre/pro-B cells, immature B cells, mature B cells probed by BrdU incorporation. **(E)** Statistical analysis of apoptosis of BM pre/pro-B cells, immature B cells and mature B cells probed by cleaved caspase 3 expression. **(F)** Statistical analysis of proliferation and apoptosis of splenic B cells probed by BrdU incorporation and active caspase 3 expression. For **(B, D–F)**, data were shown as mean +/- S.E.M. Each symbol represents one mouse. Student t test was used for statistical analysis. * p< 0.05; * p< 0.01; *** p<0.001. Experiments were repeated at least 3 times with the same pattern.

**Figure 3:**
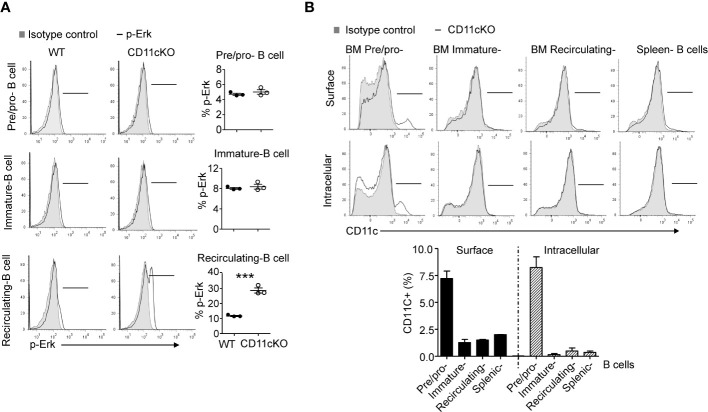
Phopho-Erk1 expression 9and CD11c expression in B cells. **(A)** Phospho-Erk expression. Left-panel: representative histogram overlay analysis of phosphor-Erk in all of pre/pro B cells, immature B cells, mature B cells in bone marrow from WT and CD11cKO mice; Right-panel: data were shown as mean +/- S.E.M. Each symbol represents one mouse. Student t test was used for statistical analysis. *** p<0.001. Experiments were repeated twice with the same pattern. **(B)** Intracellular and cell surface CD11c expression was probed in BM pre/pro B cells, immature B cells, mature B cells and splenic B cells. Up-panel: Representative histogram from 5 independent experiments was shown. Bottom-panel: Statistical analysis of one experiment. Data were shown as mean +/- S.D (n=3 per group).

We previously showed that CD11c plays a role in proliferation and apoptosis of developing neutrophils (3). Therefore, we assessed if proliferation and apoptosis was also involved in the maintenance of BM and splenic B cells in the presence or absence of CD11c. Co-staining of Ki67 and cleaved-Caspase 3 showed that, in the BM, CD11c KO recirculating B cells demonstrated significantly higher apoptosis and proliferation compared to their WT counterparts, while there was no difference in pre/pro-B cells and immature B cells between the two strains (**Figures 2A, B**), supporting our findings on their numbers. Further *in vivo* BrdU incorporation study confirmed that recirculating B cells in the BM were resting and non-proliferating cells in WT mice. However, in the absence of CD11c, re-circulating B cells lost the quiescence and entered the cell cycle robustly (**Figures 2C, D**). In addition to zealous proliferation, active caspase 3 levels alone revealed that, in the absence of CD11c, these proliferating recirculating B cells were highly pro-apoptotic in the bone marrow (**Figure 2E**). Indeed, this was not limited to BM recirculating B cells. In the spleen, we also observed that, in the absence of CD11c, mature B cells were more proliferative and apoptotic, suggesting the loss of quiescence (**Figure 2F**). To date, the survival mechanism of re-circulating B cell in the bone marrow is largely unknown. As a preliminary discovery, we investigated the involvement of Erk signaling pathway. Surprisingly, we only detect marginal signaling of phosphor-Erk in all of B cell subpopulations in both genotypes, except a significant higher phosphorylation of Erk in recirculating B cells in CD11c KO mice (**Figure 3A**). Notably, this prominent phospho-Erk level only exists in recirculating B cells, not in pre/pro-B or immature-B cells, which might be the answer for zealous proliferation and apoptosis of recirculating B cells in CD11c KO mice. When we examined CD11c expression, there was a very limited CD11c expression observed on recirculating B cell and splenic B cells (**Figure 3B**) suggesting that these phenotypes might have been driven indirectly rather than intrinsically.

### Macrophage migration inhibitory factor was significantly lower in CD11c KO bone marrow dendritic cells

**Figure 4:**
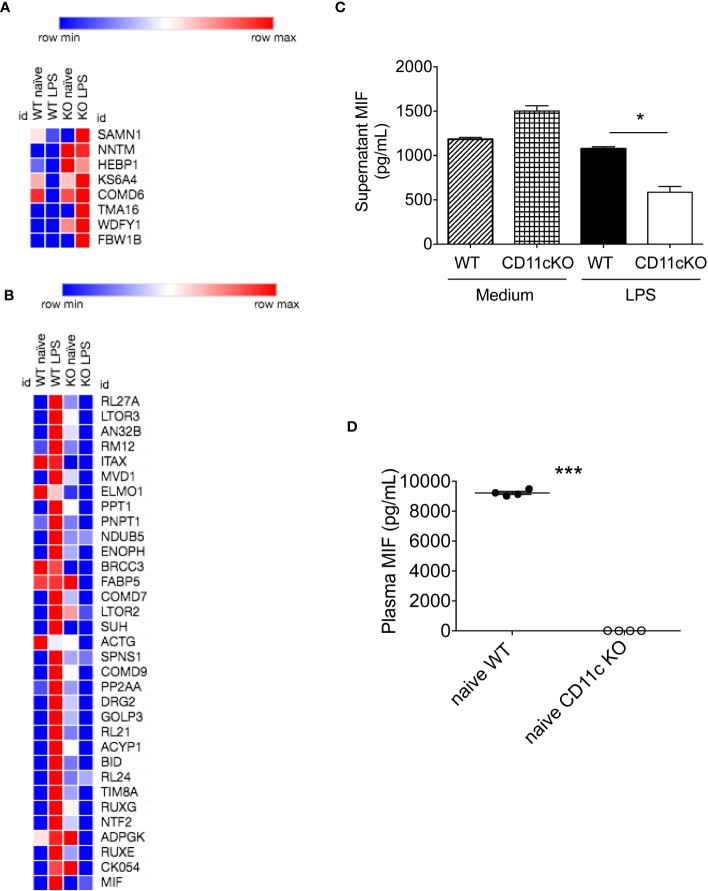
*In vitro* and *in vivo* MIF level analysis. **(A)** Heatmap of upregulated proteins was shown. Morpheus software was used to generate heatmap. **(B)** Heatmap of downregulated proteins was shown. **(C)** MIF levels in the supernatant of cultured WT and CD11c KO BMDCs were measured. Data were shown as mean +/- S.D (n=4 per group). One-way ANOVA with *post hoc* Bonferroni analysis was used for statistical analysis. * p< 0.05. **(D)** Plasma MIF levels in naïve WT and CD11c KO mice. Data were shown as mean +/- S.D. Student t test was used for statistical analysis. *** p< 0.001.

Given dendritic cells are the major leukocytes expressing CD11c, we compared WT and CD11c KO bone marrow dendritic cells (BMDCs) using proteomics with or without stimulation. We found 11 differentially upregulated proteins and 37 downregulated proteins under LPS stimulation (**Table 1**, **Figures 4A, B**). No differentially expressed proteins were noted without stimulation. We hypothesized that some of differentially expressed proteins would serve as B cell stimulants. Ontology analysis showed that these differentially expressed proteins largely represented intracellular process such as RNA binding and ribonucleoprotein (RNP) binding (**Supplementary Figure 1**). In contrast, macrophage migration inhibitor factor (MIF) can be released from the intracellular compart to extracellular place by cell such as myeloid cells (11). To confirm the proteomics finding, we cultured BMDCs and examined MIF levels in the supernatant by ELISA. In support of our prediction, we observed a significant decrease in MIF levels in the supernatant of CD11c KO BMDCs stimulated with LPS compared to WT BMDCs (**Figure 4C**). Interestingly, LPS stimulation didn’t dramatically induce MIF. In the culture with medium only, we found the level of MIFs in supernatants between WT and CD11c KO BDMCs are comparable, and CD11c KO ones even slightly higher. We do not know what regulates the half-life of MIFs in the culture media of BMDCs at this point. However, we observed limited change of MIF levels in the supernatant of CD11c KO BMDCs without stimulation. We also examined *mif* gene expression using qPCR. We did not find any difference (**Supplementary Figure 2**), indicating that CD11c might affect MIF at the level of transportation in DCs. To further confirm the role of CD11c in MIF levels *in vivo*, we measured plasma MIF levels in native WT and CD11c KO mice. MIF levels were significantly lower in native CD11c KO mice (**Figure 4D**). MIF binds to its receptor CD74 on B cells (12). The binding of MIF to CD74 activates PI3K/Akt pathway, controlling B cell proliferation and survival (13, 14). Thus, our finding showed B cell number was controlled by CD11c at least in part through DCs.

**Table 1:** The list of differentially expressed proteins in CD11c BMDCs with LPS stimulation.

Upregulated proteins	
Uniprot ID	Protein name
P57725	SAM domain containing protein SAMSN-1 (SAMN1)
Q61941	NAD(P) transhydrogenase (NNTM)
Q9R257	Hemobinding protein 1 (HEBP1)
Q9Z2B9	Ribosomal protein S6 kinase alpha-4 (KS6A4)
Q3V4B5	COMM domain containing protein 6 (COMD6)
Q9CR02	Translation machinery associated protein 16 (TMA16)
E9Q4P1	WD repeat and FYVE domain containing protein 1 (WDFY1)
Q5SRY7	F box and WD repeat domain containing 11 (FBW1B)
Downregulated proteins	
Uniprot ID	Protein name
P14115	Large ribosomal subunit protein uL15 (RL27A)
O88653	Regulator complex protein LAMTOR3 (LTOR3)
Q9EST5	Acidic leucine-rich nuclear phosphoprotein 32 (AN32B)
Q9DB15	Ribosomal protein 12 mitochondrial large ribosomal subunit. (RM12)
Q9QXH4	Integrin alpha-X (ITAX)
Q99JF5	Mevalonate diphosphate decarboxylase (MVD1)
Q8BPU7	Engulfment and cell motility 1 (ELMO1)
O88531	Palmitoyl-protein thioesterase 1 (PPT1)
Q8K1R3	Polyribonucleotide nucleotidyltransferase 1. (PNPT1)
Q9CQH3	NADPH ubiquinone oxidoreductase subunit B5. (NDUB5)
Q8BGB7	Enolase phosphatase 1. (ENOPH)
P46737	BRCA1/BRCA2 containing complex subunit 3. E3 ubiquitin ligase (BRCC3)
Q05816	Fatty acid binding protein 5 (FABP5)
Q8BG94	COMM domain containing protein 7 (COMD7)
Q9JHS3	Regulator complex protein LAMTOR2 (LTOR2)
P31266	Recombining binding protein suppressor of hairless (SUH)
P63260	Actin, cytoplasmic 2 (ACTG)
Q8R0G7	Protein spinster homolog 1 (SPNS1)
Q8K2Q0	COMM domain containing protein 9.(COMD9)
P63330	Serine/threonine-protein phosphatase 2A catalytic (PP2AA)
Q9QXB9	Developmentally regulated GTP binding protein 2 (DRG2)
Q9CRA5	Golgi phosphoprotein 3 (GOLP3)
O09167	Large ribosomal protein eL21 (RL21)
P56376	Acylphosphatase 1 (ACYP1)
P70444	BH3 interacting domain death agonist (BID)
Q8BP67	Large ribosomal subunit protein EL24 (RL24)
Q9WVA2	Mitochondrial import inner membrane translocase subunit Tim8A (TIM8A)
P62309	Small nuclear ribonucleoprotein polypeptide G. (RUXG)
P61971	Nuclear transport factor 2 (NTF2)
Q8VDL4	ADP-dependent glucokinase. (ADPGK)
P62305	Small nuclear ribonucleoprotein E (RUXE)
Q91V76	Ester hydrolase C11orf54 homolog (CK054)
P14174	Macrophage migration inhibitory factor (MIF)

## Discussion

B cells are a critical part of the adaptive immune system. The number of naïve mature B cells are carefully controlled by various factors (15). In this study, we found a novel role of CD11c in regulating B cell number.

B cell regulation by B cell activating factor (BAFF), a member of the tumor necrosis factor (TNF) has been extensively studied. It serves as a survival factor for immature and mature B cells (16, 17). BAFF overexpression leads to an expansion of B cell compartment (18). BAFF is produced by DCs, but our proteomics did not observe BAFF as differentially expressed proteins. Instead, we found MIF as differentially expressed proteins in CD11c KO DCs. MIF binds to CD74 and regulates PI3K/AKT signal for survival (13). In line with this, we observed CD11c KO recirculating and mature B cells with significantly higher proliferative and apoptotic capability. In addition to its role in B cell survival, MIF serves as a chemoattractant for B cells (19). B cells are attracted to MIF in CXCR4- and CD74-dependent manner. Thus, less splenic B cells in CD11c KO mice may also be due to less recruitment of B cells to the spleen.

MIF was one of the first cytokines to be discovered, but its role had been ambiguous for a while (20). However, its involvement in the regulation of immunity has been reported over years including B cell survival. Interestingly, the mechanism of how MIF is produced has not been completely delineated yet. It was reported that MIF would be secreted via non-classical pathway involving an ABC transporter (21), but its ultimate evidence has not been reported yet (22). Recent paper suggested MIF release was mediated by necrosis, necroptosis and pyroptosis (23). However, it is unlikely that CD11c KO cells had less cell death. Rather our data showing the involvement of CD11c in MIF release will likely add a new angle for this investigation. CD11c belongs to a critical adhesion molecule family β2 integrin consisting of four members; CD11a, CD11b, CD11c and CD11d. Previously we observed CD11c was also intracellularly expressed in DCs (3). In neutrophils, we suggested that IQ motif containing GTPase activating protein 1 (IQGAP1) serves as an intracellular ligand (3). Thus, as a future project, we will examine how CD11c regulates MIF release from DCs. Despite CD11b and CD11d are extremely homologous to CD11c (6), we did not observe any effect on B cell count by CD11b and CD11d. Thus, our data also suggested the unique feature of CD11c.

B cells are involved in a number of diseases including autoimmune diseases such as multiple sclerosis, rheumatoid arthritis, and systemic lupus erythematosus (24). B cells enhance immune response in autoimmunity by producing autoantibody producing plasma cells and promoting CD4+ T cell responses. Plasma cells represent the terminal differentiation step of mature B cells (24). We showed that CD11c deficiency lowered the number of mature B cells, suggesting that it might be possible to reduce the production of autoantibody. Further workup needs to be performed to determine the role of CD11c in this aspect.

In conclusion, we demonstrated a novel mechanism of CD11c mediated B cell regulation.

## Data availability statement

The mass spectrometry proteomics data in the study have been deposited to the ProteomeXchange Consortium (https://proteomecentral.proteomexchange.org) via the iProX partner repository with the dataset identifier PXD048907.

## Ethics statement

The animal study was approved by Institutional Animal Care and Use Committee of Boston Children’s Hospital. The study was conducted in accordance with the local legislation and institutional requirements.

## Author contributions

LH: Writing – original draft, Writing – review & editing. Y-CS: Formal Analysis, Investigation, Writing – original draft, Writing – review & editing. YC: Investigation, Writing – review & editing, Writing – original draft. KY: Formal Analysis, Funding acquisition, Investigation, Writing – original draft, Writing – review & editing.

## Supplementary material

Supplementary Figure 1 Ontology analysis of differentially expressed proteins in CD11c KO BMDCs. Differentially expressed proteins were applied to KEGG analysis. **(A)** GO-Biological processes of upregulated proteins. **(B)** GO-Biological processes of downregulated proteins.

Supplementary Figure 2 RT-qPCR analysis of both interferon-beta and MIF in BMDC. BMDCs with or without LPS stimulation were subjected to RT-qPCR analysis. GAPDH was used for reference gene.
