# Supplementary figures and images for: Integrin CD11c regulates B cell homeostasis
Source: Front Immunol. 2024 Feb 6;15:1359608. doi: 10.3389/fimmu.2024.1359608 (PMC10876775; doi:10.3389/fimmu.2024.1359608)

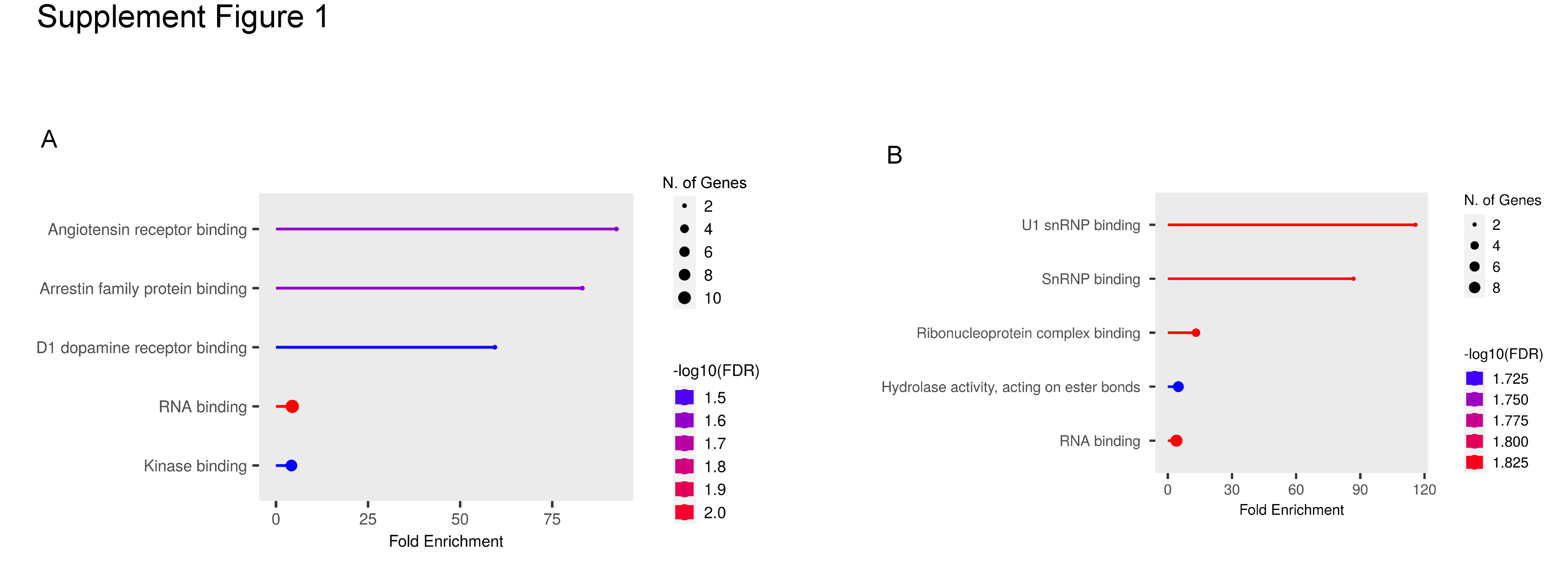

Supplement: Supplementary Figure 1 — Ontology analysis of differentially expressed proteins in CD11c KO BMDCs. Differentially expressed proteins were applied to KEGG analysis. (A) GO-Biological processes of upregulated proteins. (B) GO-Biological processes of downregulated proteins. [file Image_1.tif]

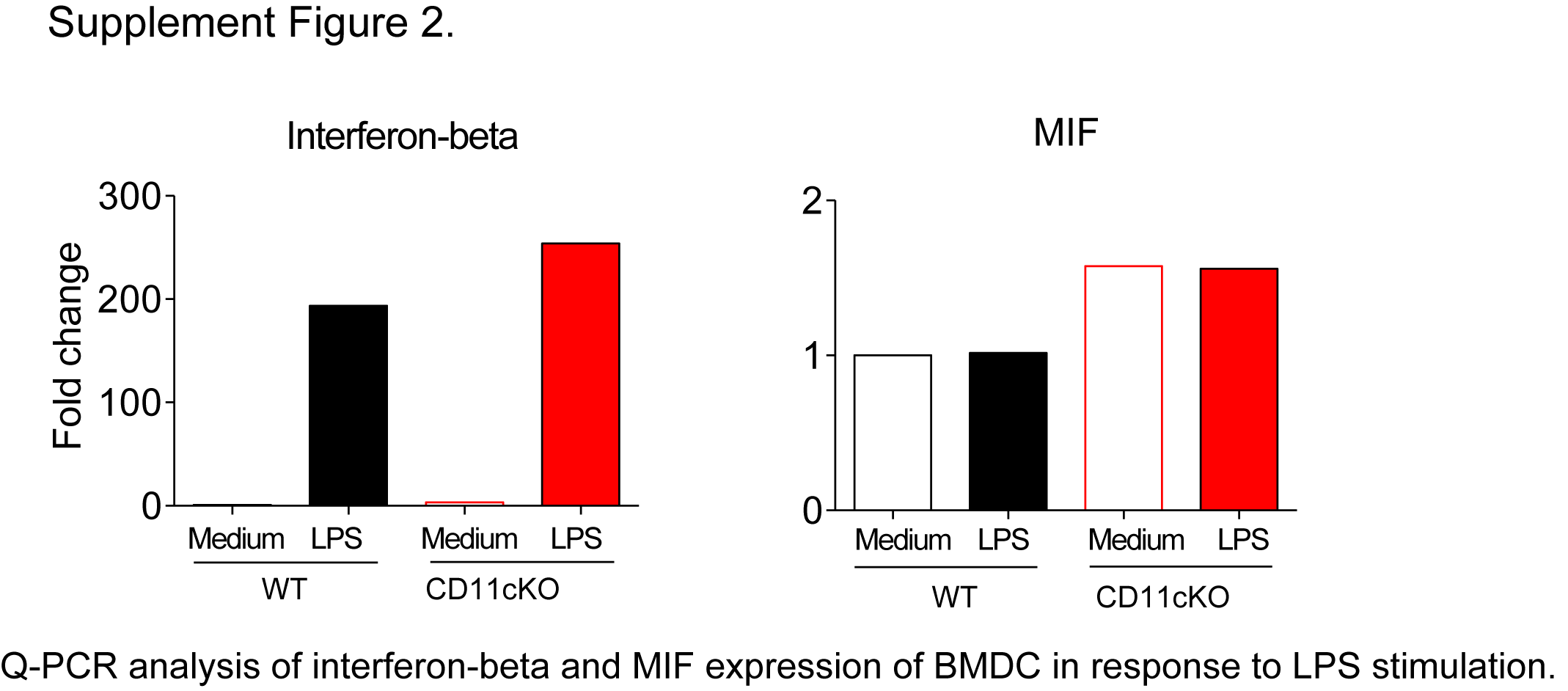

Supplement: Supplementary Figure 2 — RT-qPCR analysis of both interferon-beta and MIF in BMDC. BMDCs with or without LPS stimulation were subjected to RT-qPCR analysis. GAPDH was used for reference gene. [file Image_2.tif]
